# Anticancer effects of 3,3′-diindolylmethane are associated with G1 arrest and mitochondria-dependent apoptosis in human nasopharyngeal carcinoma cells

**DOI:** 10.3892/ol.2012.1063

**Published:** 2012-12-05

**Authors:** YU XU, JIN ZHANG, WENDAN SHI, YUN LIU

**Affiliations:** 1 Departments of Otolaryngology, Wuhan University, Wuhan 430060, Hubei, P.R. China; 2 Gastroenterology, Renmin Hospital, Wuhan University, Wuhan 430060, Hubei, P.R. China

**Keywords:** 3,3′-diindolylmethane, nasopharyngeal carcinoma, cell cycle arrest, apoptosis, mitochondria

## Abstract

The antitumor effects of 3,3′-diindolylmethane (DIM) are exhibited in a number of human cancer cells. However, there have been few studies performed concerning the effect of DIM on nasopharyngeal cancer (NPC) cells. In the present study, we examined the *in vitro* antitumor activity of DIM on the poorly differentiated NPC cell line CNE-2. The potential molecular mechanisms of the activity were also explored. CNE-2 cells were treated with varying concentrations of DIM for different times. Cell proliferation and apoptosis were detected and the molecular mechanisms involved in these effects were characterized. The results demonstrated that DIM at concentrations of 15–100 *μ*M caused dose- and time-dependent inhibition of CNE-2 cell proliferation. Flow cytometry analysis revealed a high sub-G1 cell peak following treatment with DIM, and the rate of apoptosis increased. DIM may elevate the levels of cleaved Bid and Bax and enhance mitochondrial membrane depolarization, allowing the efflux of cytochrome c, Smac and Omi into the cytosol. The levels of caspases-3, -8 and -9 and cleaved poly (ADP-ribose) polymerase (PARP) were upregulated following DIM treatment in a dose-dependent manner. DIM also inhibits the phosphorylation of IκB-α, and showed dose-dependent inhibition of Bcl-2, XIAP and NF-κB in CNE-2 cells *in vitro*. These results indicate that DIM inhibits cell proliferation by inducing cell cycle arrest at G0/G1 phase and induces the apoptosis of CNE-2 cells by regulating multiple molecules in a mitochondria-dependent pathway. DIM may be a preventive and therapeutic agent against NPC.

## Introduction

Nasopharyngeal carcinoma (NPC) is one of the most common cancers of the head and neck, particularly in Southern China and Southeast Asia, where the annual incidence is >20 cases per 100,000 individuals (1,2). In Guangzhou province in China, the annual incidence reaches 25 cases per 100,000 (3). A combination of radiotherapy and adjuvant chemotherapy is now the standard treatment for NPC, and these treatment modalities have been successful for certain patients. However, the 5-year survival rate is only 50–60% due to the frequency of distant metastasis and local recurrence, as well as long-term secondary effects of radiotherapy and chemotherapy. Additionally, there is almost no effective treatment for those who are resistant to radiotherapy and have tumor recurrence. There is an urgent need to develop new treatment strategies for this patient population, which will be both better tolerated and more effective.

Recently, much attention has been focused on the use of dietary phytochemicals in cancer prevention and therapy due to the pleiotropic effects of these agents on multiple carcinogen-activated oncogenic pathways, and their equally excellent safety profiles (4,5). Among various natural products, indole-3-carbinol (I3C) has been reported to inhibit cancer development by perturbing multiple cellular signaling events (6). In our previous research, we found that I3C induced mitochondria-mediated apoptosis in the NPC cell line CNE-2 (7). In the stomach, I3C is rapidly converted to a variety of condensation products, chiefly 3,3′-diindolyl-methane (DIM) (8). Researchers have also found that DIM spontaneously formed from I3C during cell culture experiments (9). Therefore DIM, rather than I3C, may be the major compound available to cells following ingestion of I3C. It has been shown that DIM inhibits the proliferation of a variety of cancer cell types, including prostate (10,11), breast (12,13) and colon (14) cancer cells, as well as certain cervical cancer cell lines (15), via induction of cell cycle arrest and apoptosis. Since susceptibility to an agent may differ among different cancer cells, it has remained unclear whether DIM has similar effects on human NPC cells. In the present study, we investigated the effects of DIM on CNE-2, a poorly differentiated human NPC cell line that is the most common cell type in NPC. The possible pathways and molecular mechanisms involved in its effects were also explored.

## Materials and methods

### Reagents and antibodies

DIM, dimethyl sulfoxide (DMSO), propidium iodide (PI), bovine serum albumin (BSA) and Triton X-100 were purchased from Sigma Company (St. Louis, MO, USA). The mitochondrial membrane potential detection kit was purchased from Stratagene (Cedar Creek, TX, USA). The respective antibodies against caspase-3, -8, -9, Bid, Bax, Bak, Bcl-2, c-FLIP, cleaved caspase-3, -8, -9 and poly (ADP-ribose) polymerase (PARP) were purchased from Cell Signaling Technology, Inc. (Beverly, MA, USA). Antibodies against NF-κB, NF-κB p65 and p50, cyclin A, cyclin D1, cyclin E, cyclin-dependent kinase (CDK)1, CDK2, p53, p21, p27, cytochrome c, Smac, Omi, and GAPDH were obtained from Santa Cruz Biotechnology (Santa Cruz, CA, USA). The mouse monoclonal anti-X-linked apoptosis-inhibiting protein (XIAP) antibody was purchased from BD Biosciences Pharmingen, Inc. (San Jose, CA, USA). The anti-IκB-α (pS32) phosphospecific antibody was purchased from Cell Signaling Technology (Danvers, MA, USA). Anti-IKK-β and anti-p-IKK-β antibodies were purchased from Imgenex (San Diego, CA, USA). The study was approved by the ethics committee of Renmin Hospital of Wuhan University, Wuhan, China.

### Cell culture

CNE-2, a poorly differentiated human NPC cell line, was obtained from the Type Culture Collection of the Chinese Academy of Sciences (Shanghai, China) and cultured in RPMI-1640 medium (Gibco Laboratories, Grand Island, NY, USA), supplemented with 10% fetal bovine serum (Sijiqing Biological Engineering Company, Hangzhou, China) and 1% penicillin/streptomycin in a humidified atmosphere of 5% CO_2_ at 37°C.

### Cell viability assay

CNE-2 cells were treated with DMSO or different concentrations of DIM (0, 15, 30, 50 and 100 *μ*M) for 6, 12, 24 or 48 h. For the methylthiazolyldiphenyl-tetrazolium (MTT) assay, 0.5 mg/ml MTT (pH 4.7; Sigma) was added 4 h before the end of the culture time. After the supernatant was discarded, MTT formazan precipitates were dissolved in 150 *μ*l DMSO, shaken mechanically for 10 min and then read immediately at 570 nm in a plate reader. Wells without cells were used as blanks. Three independent experiments were performed.

### Cell proliferation assay

The inhibition of [^3^H]-TdR incorporation into DNA was examined using the pulse-labeling method. Proliferating CNE-2 cells were seeded into 96-well plates and incubated for 16 h and then treated with DMSO or different concentrations of DIM (0, 15, 30, 50 and 100 *μ*M) for the next 6, 12, 24 or 48 h. For the [^3^H]-TdR assay, CNE-2 cells were exposed to [^3^H]-TdR for 6 h. After the cells were harvested onto glass fiber filters, the counts per minute (cpm) were measured with a liquid scintillation counter (FJ-2107P, Xi’an, China). Three independent experiments were performed.

### Cell cycle analysis by flow cytometry

Cells were grown in the absence or presence of DIM (15, 30, 50 or 100 *μ*M) or presence of DMSO for 48 h. For cell cycle analysis, cells were collected, washed twice with 0.01 M phosphate-buffered saline (PBS) and fixed in 70% ethanol overnight at 4°C. The cells were subsequently washed in PBS, digested with 200 *μ*l RNase (1 mg/ml) at 37°C for 30 min and stained with 800 *μ*l PI (50 *μ*g/ml) at room temperature for 30 min. The cells were then washed with PBS and immediately analyzed using flow cytometry. The percentages of nuclei in CNE-2 cells in each phase of the cell cycle (G1, S and G2/M) were calculated using the MultiCycle software program (Phoenix Flow Systems, San Diego, CA, USA).

### Apoptosis assays by flow cytometry

To assay the effect of DIM on cell apoptosis, CNE-2 cells were exposed to DMSO or 0, 15, 30, 50 or 100 *μ*M DIM for 48 h. The cultured cells were trypsinized and 1×10^6^ cells were collected by centrifugation. After washing with PBS and centrifuging at 1,000 × g for 5 min, the cell pellet was resuspended in Annexin V-FITC/PI and finally analyzed with a FACSort flow cytometer (Becton-Dickinson, Mountain View, CA, USA).

### Analysis of mitochondrial membrane potential

Mitochondrial membrane potential (Δψm) was measured using the mitochondrial membrane potential detection kit. Briefly, cells were stained with Rh123 for 15 min and then measured on a FACScan flow cytometer according to the instruction manual.

### Western blot analysis

Following treatment with 0, 15, 30, 50 or 100 *μ*M DIM for 48 h, cells were washed with PBS, harvested and lysed in RIPA buffer [50 mM Tris-HCl (pH 8.0) with 150 mM NaCl, 1.0% Nonidet P-40, 0.5% sodium deoxycholate and 0.1% sodium dodecyl sulfate] containing protease inhibitor and phosphatase inhibitor (Roche, Indianapolis, IN, USA). Protein concentration was determined using the BCA reagent (Pierce, Rockford, IL, USA). Equal amounts of protein were subjected to sodium dodecyl sulfate-polyacrylamide gel electrophoresis and transferred to a polyvinylidene difluoride membrane. After blocking with 5% BSA in TBS at room temperature for 1 h, membranes were incubated with an appropriate dilution of primary antibody at 4°C overnight. Membranes were washed extensively with 0.1% Tween-20 in TBS and incubated with secondary antibody at room temperature for 1 h. Bands corresponding to the antibodies were detected by enhanced chemiluminescence (Pierce) and quantified using Quantity One imaging software (Bio-Rad, Hercules, CA, USA).

### NF-κB DNA binding activity measurement

CNE-2 cells were plated in 100-mm dishes at a density of 1×10^6^ cells and cultured. After 24 h, the cultures were treated with DMSO or DIM (0, 15, 30, 50 or 100 *μ*M) for 6, 12, 24 and 48 h. Following treatment, cells were resuspended in 10 mM Tris-HCl (pH 7.5)/5 mM MgCl_2_/0.05% Triton X-100 and lysed with a homogenizer. The homogenate was centrifuged at 3,000 × g for 15 min at 4°C. The nuclear pellet was then resuspended in an equal volume of 10 mM Tris-HCl (pH 7.4)/5 mM MgCl_2_ followed by the addition of one nuclear pellet volume of 1 M NaCl/10 mM Tris-HCl (pH 7.4)/4 mM MgCl_2_. The lysing nuclei were put on ice for 30 min before centrifugation at 10,000 × g for 15 min at 4°C. The supernatant (nuclear extract) was removed and the protein concentration was measured using the BCA protein assay. To determine NF-κB DNA binding activation, electrophoretic mobility shift assay (EMSA) was carried out. Briefly, the NF-κB oligonucleotide 5′-AGTTGAGGGGACTTTCCCAGGC-3′ DNA-binding sequence was labeled by the Biotin 3′ end labeling kit, and 10 *μ*g nuclear proteins were incubated for 20 min at room temperature with biotin-labeled DNA probes in the 20 *μ*l shift-binding buffer comprising 25 mM EDTA, 5 mM MgCl_2_, 0.05% NP-40, 2.5% glycerol, 50 ng/*μ*l poly(dI·dC) and 0.2 *μ*g BSA. Nucleo-protein complexes were loaded onto the pre-electrophoresis 6% non-denaturing polyacrylamide gels in 0.5X Tris-boric acid-EDTA buffer at 100 V for 2 h at room temperature. The electrophoresed binding reactions were transferred to a nylon membrane by capillary transfer system overnight at room temperature. The signal was detected using the conjugated streptavidin-horseradish peroxidase and chemiluminescent substrate and quantified with Quantity One imaging software (Bio-Rad).

### Statistical analysis

All experiments were repeated at least three times independently, and values are expressed as the mean ± standard deviation (SD). Analysis of variance (ANOVA) with subsequent Bonferroni’s test was used to determine significant differences in multiple comparisons. Values of P<0.01 were considered to indicate a statistically significant result.

## Results

### Effects of DIM on the viability and proliferation of CNE-2 cells

**Figure 1.**
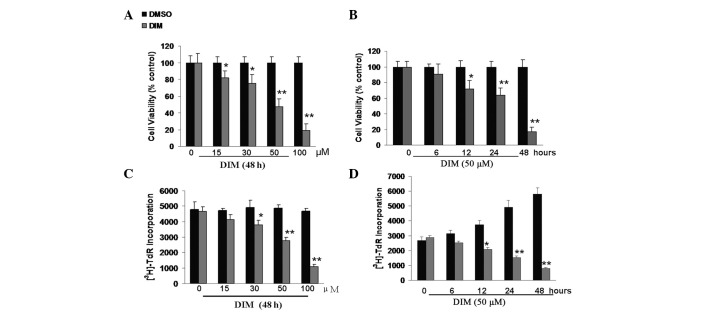
Effects of 3,3′-diindolylmethane (DIM) on cell growth in CNE-2 cells. The results are shown as the mean viable cell ratio ± SD from three independent experiments. ^*^P<0.05, ^**^P<0.01 compared with 0 *μ*M DIM or dimethyl sulfoxide (DMSO) treatment. For dose-dependent analysis, 2×10^4^ cells were cultured with DMSO and 0, 15, 30, 50 or 100 *μ*M DIM in 96-well plates for 48 h. For time-dependent analysis, 2×10^4^ CNE-2 cells were cultured in 96-well plates for different time periods in the presence of 50 *μ*M DIM. (A and B) The effect of DIM on the viability of CNE-2 cells measured by the MTT assay: (A) dose-dependent analysis and (B) time-dependent analysis. (C and D) The effect of DIM on proliferation of CNE-2 cells. The pulse-labeling method was used to detect [^3^H]-TdR incorporation into DNA. The results revealed that treatment with DIM also induced growth inhibition of CNE-2 in a dose- and time-dependent manner.

CNE-2 cells were exposed to DMSO and various concentrations of DIM for 48 h or 50 *μ*M of DIM for 6–48 h. The MTT assay was used to examine the effect of DIM on cell viability. Results showed that DIM inhibited CNE-2 cell viability in a time- and dose-dependent manner. When exposed for 48 h, the viability of CNE-2 cells was significantly inhibited at concentrations of 15–100 *μ*M DIM (Fig. 1A). At a concentration of 50 *μ*M, DIM caused a significant decrease in the viability of CNE-2 cells at exposure times of 12 h or longer (Fig. 1B).

To examine the effect of DIM on the proliferation of CNE-2 cells, we used the pulse-labeling method to detect [^3^H]-TdR incorporation into DNA. Treatment with DIM also induced growth inhibition of CNE-2 in a dose- and time-dependent manner. Concentrations of 30–100 *μ*M DIM were able to inhibit the growth of CNE-2 at 48 h (Fig. 1C), and even a 12 h exposure to 50 *μ*M DIM significantly decreased the proliferation of CNE-2 cells (Fig. 1D).

### DIM induces cell cycle arrest at the G1 phase and reduces the levels of CDKs, CDK inhibitors (CDKIs) and cyclin proteins

**Figure 2.**
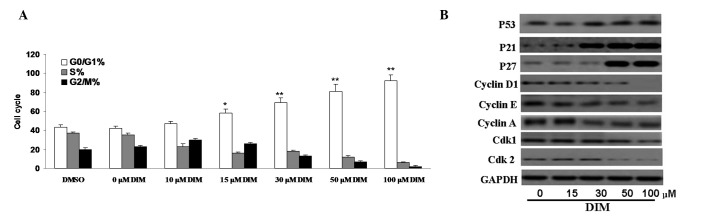
Effect of 3,3′-diindolylmethane (DIM) on the cell cycle of CNE-2 cells. (A) Treatment of cells with various concentrations of DIM for 48 h resulted in dose-dependent increases in the percentage of cells in G0/G1 phase. The percentage of CNE-2 cells arrested in the G0/G1 phase was much higher in the DIM-treated group than in the dimethyl sulfoxide (DMSO) or untreated groups (^*^P<0.05, ^**^P<0.01). (B) Proteins controlling the phase of the cell cycle, such as CDK1, CDK2, cyclin D1, cyclin A and cyclin E, were found to be downregulated by treatment with DIM using western blot analysis. Meanwhile, expression levels of CDK1, as well as p53, p27 and p21, were decreased in the presence of DIM.

It has been shown that I3C induces G1 cell cycle arrest in breast cancer cells (13). To determine whether DIM regulates cell cycle progression in CNE-2 cells, the DNA was stained with PI, then FACS analysis was performed. The results showed that the percentage of CNE-2 cells arrested in the G0/G1 phase was markedly higher in the 15 *μ*M DIM-treated group than in either the DMSO or untreated groups (59.2±1.9 vs. 44.3±1.0 and 42.4±0.4%, respectively, P<0.05). Treatment of cells with DIM for 48 h resulted in a dose-dependent increase in the percentage of cells in the G0/G1 phase, with a concomitant reduction in cell numbers in the S phase (Fig. 2A).

To confirm cell cycle arrest, we further examined cell cycle regulatory molecules in DIM-treated CNE-2 cells. Our results showed that the levels of CDK2 and CDK1 proteins in CNE-2 cells were reduced significantly following treatment with 50–100 *μ*M DIM for 48 h. The levels of cyclin D1, cyclin A and cyclin E were also significantly suppressed following treatment with 50–100 *μ*M DIM. The expression levels of CDKIs involved in cell cycle arrest, such as p21 and p27, were increased in a time-dependent manner in cells treated with DIM. Thus, DIM induces cell cycle arrest by affecting the expression of multiple cyclin-related cellular signaling proteins (Fig. 2B).

### DIM-induced apoptosis is accompanied by mitochondrial changes and mitochondria-related protein alterations

**Figure 3.**
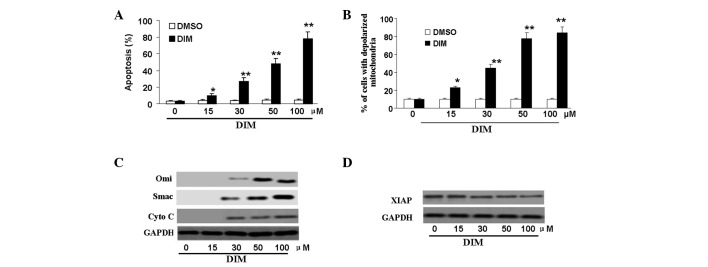
Effect of 3,3′-diindolylmethane (DIM) on apoptosis and expression of mitochondria-related apoptotic proteins in CNE-2 cells. (A) Effect of DIM on apoptosis of CNE-2 cells, estimated by flow cytometry. The percentage of apoptotic CNE-2 cells was markedly higher in the DIM-treated group than in the dimethyl sulfoxide (DMSO) or untreated groups (^*^P<0.05, ^**^P<0.01, DIM-treated group vs. DMSO-treated group vs. untreated group). (B) Treatment of cells with various concentrations of DIM for 48 h resulted in dose-dependent increases in the percentage of cells with depolarized mitochondria (^*^P<0.05, ^**^P<0.01). (C) Expression of cytochrome c, Smac and Omi in DIM-treated CNE-2 cells after 48 h. The release of cytochrome c, Smac and Omi was shown to be increased in a dose-dependent manner by DIM using western blot analysis. (D) Western blot analysis revealed that the level of XIAP was decreased in a dose-dependent manner by DIM.

To determine whether DIM inhibited CNE-2 cell viability by inducing apoptosis, flow cytometric analysis was carried out using double-staining with Annexin V-FITC and PI. CNE-2 cells were treated with DMSO or DIM at the indicated concentrations for 48 h. The results showed that apoptotic cells were markedly increased following treatment with DIM. DIM induced apoptosis in a dose-dependent manner. A concentration of 15 *μ*M DIM significantly increased the percentage of apoptotic cells at 48 h according to densitometry analysis (P<0.05); when the concentration of DIM was 30 *μ*M, the numbers of apoptotic cells were increased almost 10-fold compared with those in DMSO-treated cells (Fig 3A).

To explore the mechanisms by which DIM induces apoptosis, we evaluated the role of mitochondria in DIM-treated CNE-2 cells. Specifically, Δψm was examined by flow cytometry. As shown in Fig. 3B, a dose-dependent increase in the level of mitochondrial membrane depolarization was observed.

Since changes in mitochondrial membrane potential are usually associated with increased permeability of the outer mitochondrial membrane, allowing efflux of apoptogenic proteins to the cytosol (16), we measured the release of cytochrome c, Smac and Omi from the mitochondria to the cytosol by western blot. As expected, the expression of cytochrome c, Smac and Omi proteins in the cytoplasm increased in a dose-dependent manner (Fig. 3C); when the concentration of DIM was 30 *μ*M, the effects were significant (P<0.01). A previous study had demonstrated that cytosolic Smac could bind XIAP and neutralize its anti-apoptotic activity (17). In this study, we further demonstrated that treatment with DIM at 30–100 *μ*M decreased the expression of XIAP (P<0.05; Fig. 3D).

### Effects of DIM on caspase protein and apoptotic protein levels

**Figure 4.**
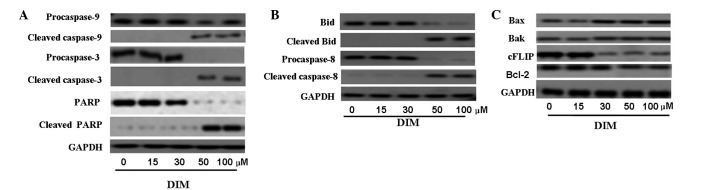
Effects of 3,3′-diindolylmethane (DIM) on apoptosis proteins in CNE-2 cells after 48 h. The proteins were identified by western blot analysis and normalized by GAPDH. (A) Increased levels of cleaved caspase-9, -3 and PARP proteins following treatment with 50 *μ*M DIM for 48 h were observed. (B) Cleaved forms of Bid and caspase-8 were also observed to be increased after 48 h treatment with 50 *μ*M DIM. (C) Anti-apoptotic proteins, such as Bcl-2 and c-FLIP, were found to be downregulated by treatment with DIM, while pro-apoptotic proteins, such as Bax and Bak, were upregulated at 48 h. PARP, poly (ADP-ribose) polymerase.

Caspases are important regulators of cell apoptosis. Caspases -8 and -9 are the initiator caspases, while caspase-3 is the ‘executioner enzyme’. To better characterize the pathway through which DIM exerts its apoptotic effects in CNE-2 cells, we examined the effects of DIM on the levels and activities of caspases. CNE-2 cells were treated with 0, 15, 30, 50 or 100 *μ*M DIM for 48 h. The western blot results shown in Fig. 4 indicate that 50 *μ*M DIM significantly increased cleavage of caspase -9, -8 and -3 after 48 h of treatment and that this activation was strengthened by 100 *μ*M DIM. The increases in cleaved caspase-9, -8 and -3 were associated with decreases in procaspase-9, -8 and -3. As expected, an increase in cleaved PARP, a substrate of caspase 3, was also observed with 50 *μ*M DIM treatment. Bid, well known as a linker between the endogenous mitochondrial pathway and death receptor mediated extrinsic apoptotic pathway, was also activated by DIM.

Mitochondrial membrane depolarization can result from the action of pro-apoptotic and/or anti-apoptotic members of the Bcl-2 family (18). Thus, we examined the effects of DIM on Bcl-2, Bak and Bax protein expression as well as c-FLIP which is an inhibitor of apoptosis mediated by the death receptors Fas, DR4 and DR5. The results of western blot analysis showed that DIM suppressed the expression of anti-apoptotic proteins such as Bcl-2 and cFLIP and increased the levels of pro-apoptotic proteins such as Bax and Bak in a dose-dependent manner (Fig. 4C).

### DIM suppresses NF-κB activation in a dose- and time-dependent manner

**Figure 5.**
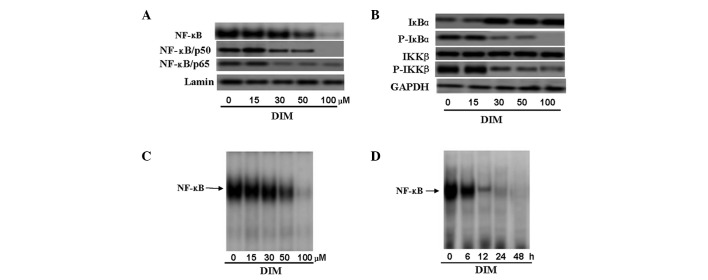
Effect of 3,3′-diindolylmethane (DIM) on NF-κB in CNE-2 cells. (A) Western blot analysis of nuclear NF-κB and p65 and p50 subunit proteins in CNE-2 cells showed that DIM (30–100 *μ*M) suppressed nuclear NF-κB expression and also decreased expression of the p50 and p65 subunits of NF-κB; these effects occurred in a dose-dependent manner. (B) Cells were incubated with different concentrations of DIM (0, 15, 30 50, and 100 *μ*M) for 48 h, and cell extracts were prepared to check the levels of IκB-α, p-IκB-α, IKK-β and p-IKK-β by western blot analysis as described in Materials and methods. All samples were probed with GAPDH to show equal protein loading. Phosphorylation of IκB-α, which can activate NF-κB, was suppressed by DIM, while decreased levels of p-IKK-β were found following treatment with DIM. (C and D) To further confirm the effect of DIM on activation of NF-κB in CNE-2 cells, nuclear proteins from cultured cancer cells treated with DIM were subjected to analysis for NF-κB DNA binding activity as measured by electrophoretic mobility shift assay (EMSA). (C) For the dose-dependent analysis, cells were incubated with different concentrations of DIM (0, 15, 30, 50 or 100 *μ*M) for 48 h and then subjected to EMSA. The results showed that 50 *μ*M inhibited NF-κB DNA binding activity in CNE-2 cells significantly (P<0.01) and DIM inhibited NF-κB DNA binding activity in a dose-dependent manner. (D) For time-dependent analysis, cells were incubated with 50 *μ*M DIM for 0, 6, 12, 24 or 48 h. The results showed that NF-κB DNA binding activity was significantly suppressed at 12 h (P<0.01) and DIM inhibited NF-κB DNA binding activity in a time-dependent manner.

NF-κB is a key regulator and transcription factor of genes that mediate apoptotic signaling pathways; it also plays critical roles in cell proliferation. We therefore strongly suspected that inactivation of NF-κB would be involved in the apoptotic pathways of CNE-2 cells treated with DIM. Western blot analysis for nuclear NF-κB and its p65 and p50 subunit proteins in CNE-2 cells showed that DIM (30–100 *μ*M) suppressed nuclear NF-κB expression and also decreased expression of its p50 and p65 subunits, and these effects occurred in a dose-dependent manner (Fig. 5A).

To further confirm the effect of DIM on the activation of NF-κB in CNE-2 cells, nuclear proteins from cultured cancer cells treated with DIM were subjected to analysis for NF-κB DNA binding activity as measured by EMSA. We found that DIM significantly inhibited the NF-κB DNA binding activity of CNE-2 cells in a time- and dose-dependent manner (Fig. 5C and D). Following treatment with 50 *μ*M DIM for 12 h, NF-κB DNA binding activity was significantly suppressed (P<0.01).

### DIM inhibited IKK activation and IκB-α phosphorylation

To further investigate DIM-induced downregulation of the activity of the NF-κB pathway, we analyzed the phosphorylation of IκB-α by western blot analysis. CNE-2 cells exposed to DIM exhibited a decrease in IκB-α phosphorylation (Fig. 5B). This finding suggested that DIM could interfere with IκB-α phosphorylation and thereby prevent its degradation, thus blocking the nuclear translocation of NF-κB.

We further tested the effects of DIM on the expression levels of IKK proteins by western blot analysis using anti-p-IKK-β and anti-IKK-β antibodies, which are required for phosphorylation of IκB-α. The results showed that DIM had no effect on the expression of IKK-β, but decreased the p-IKK-β protein level in a dose-dependent manner (Fig. 5B).

## Discussion

To obtain better clinical results in the treatment of NPC, it is essential to identify novel therapeutic agents that have less toxicity. Recently, dietary chemopreventive phytochemicals have attracted interest from numerous researchers. Epidemiological studies have shown that diets rich in fruits and vegetables are associated with a lower risk of cancer (18). A number of studies have demonstrated a decreased incidence of various types of cancer in individuals who consume large amounts of cruciferous vegetables, such as broccoli, cabbage and cauliflower (19). These vegetables were found to contain glucobrassicin, which undergoes hydrolysis by cooking (20). The main hydrolysis product of glucobrassicin is I3C. I3C can be converted into a number of polymeric products, of which DIM is the main one (21). It has been reported that DIM inhibits the growth of many cancers in *in vivo* or *in vitro* studies (10–14). However, an antitumor effect of DIM in human nasopharyngeal carcinoma, one of the most common cancers in Southern China, has not yet been thoroughly reported.

Dysregulation of proliferation and apoptosis are linked to the development of most cancers. In this study, we have demonstrated that DIM significantly decreased cell proliferation in CNE-2 cells in a dose- and time-dependent manner. We found that the inhibitory effect of DIM on the growth of CNE-2 cells may result from G0/G1 cell cycle arrest. In recent research, Choi *et al* found that DIM inhibited HT-29 human colon cancer cells and was able to induce cell cycle arrest with 10–30 *μ*M DIM, which is consistent with our results (22). This result was strengthened by our examination of proteins controlling the cell cycle phase transition. Using western blot analysis, we found that DIM reduced the levels of the CDK1, CDK2, cyclin A, cyclin D1 and cyclin E proteins at 48 h in a dose-dependent manner. Meanwhile, the apoptotic effect of DIM in CNE-2 cells was analyzed using a dual staining approach with PI and Annexin V. Our findings revealed that apoptosis of CNE-2 cells was increased in the DIM-treated groups. These findings were consistent with those of previous research and provided further support for the anticancer effect of DIM. Self-sufficiency in growth signals and escaping from programmed cell death are the main changes in cell physiology necessary to promote malignant growth (23). Therefore, a bioactive agent such as DIM, which has the ability to inhibit cell cycle progression and induce apoptosis in NPC cells, may potentially be utilized as a chemopreventive agent for NPC.

In the present study, we also attempted to explore the mechanism of DIM-induced apoptosis in CNE-2 cells. Apoptosis is a programmed cell death caused by a group of cysteine proteases known as caspases. There are two major pathways in caspase cascade activation: the extrinsic (death receptor) and the intrinsic (mitochondrial) pathways. In the extrinsic (death receptor) pathway, caspase-8 and -10 are activated following the recruitment of Fas-associated death domain (FADD) protein and death domain (DD) binding. In the intrinsic pathway, cytochrome c is released from mitochondria in response to a variety of apoptotic stimuli. The release of cytochrome c induces the cleavage of caspase-9, which contributes to the activation of effector caspases such as caspase-3 (24). The effector caspases cleave a set of vital proteins such as PARP and eventually lead to apoptosis (25).

Mitochondrial dysfunction is an important characteristic of apoptotic cell death (26,27), particularly in the intrinsic pathway. In the present study, we examined perturbations in mitochondrial membrane potential under DIM treatment. We showed that changes in CNE-2 cells associated with apoptosis were accompanied by a loss of mitochondrial membrane potential. We also found that DIM treatment resulted in the release of cytochrome c, Smac and Omi into the cytosol and activation of caspase-9 and -3 in a dose-dependent manner. From these results, we can conclude that the intrinsic pathway is involved in DIM-induced apoptosis of CNE-2 cells.

Bcl-2 has been shown to form membrane pores involved in the homeostasis of cell organelles, inhibiting the mitochondrial permeability transition and cytochrome c release, thereby functioning to block apoptosis (28,29). The ratio of pro- to anti-apoptotic molecules such as Bcl-2 and Bax is considered to be a determinant for mitochondria-related apoptosis. In the present study, we found that DIM downregulated Bcl-2 and upregulated Bax in CNE-2 cells.

In this study, we found that DIM also increased the levels of cleaved caspase-8 and Bid. Bid, a BH3 domain-containing pro-apoptotic Bcl-2 family member, is a specific substrate of caspase-8 in the extrinsic apoptotic signaling pathway. It is well known as a linker between the endogenous mitochondrial pathway and the death receptor-mediated extrinsic apoptotic pathway. Full-length Bid is inactive and localized in the cytosol, while cleaved Bid translocates to the mitochondria and transduces apoptotic signals from the cytoplasm to the mitochondria, increasing mitochondrial membrane permeability and the release of apoptosis-associated mitochondrial proteins. FLIP is an important antiapoptotic protein of the FAS-related apoptotic pathway that blocks the activation of caspase-8. In the present stud, FLIP was also found to be decreased in DIM-treated CNE-2 cells. Therefore, mitochondria-dependent apoptosis may be one of the major mechanisms by which DIM induces apoptosis in CNE-2 cells. It is possible that both the extrinsic and intrinsic pathways are involved in DIM-mediated apoptosis of CNE-2 cells.

NF-κB is one of the transcription factors regulating the expression of numerous genes critical for cell survival. It plays critical roles in the control of cell proliferation and apoptosis, as well as tumor invasion, metastasis, drug resistance and the stress response, by regulating the cell cycle, inhibiting caspase activation, removing harmful oxygen radicals and defending mitochondrial function (30). It has been reported that inactivation of NF-κB makes cells more sensitive to apoptosis-inducing agents (31). Under non-stimulating conditions, NF-κB is sequestered in the cytoplasm through tight association with the NF-κB inhibitory-protein IκB-α. In this study, we found by western blot analysis that nuclear NF-κB expression in CNE-2 cells was inhibited by DIM (50–100 *μ*M) in a dose-dependent manner. Using EMSA, we further demonstrated that the DNA binding activity of NF-κB in CNE-2 cells was significantly suppressed by DIM in a time- and dose-dependent manner. Expression of the p50 and p65 subunits of NF-κB was also suppressed in DIM-treated CNE-2 cells. Thus, DIM inhibited NF-κB activation, resulting in the downregulation of transcription of genes downstream of NF-κB such as Bcl-2 and FLIP that counteract the action of pro-apoptotic proteins including Bid, Bad and Bax, thus leading to the promotion of cell apoptosis and inhibition of cell growth. The findings suggest that inhibition of NF-κB may be an important mechanism for the anti-proliferative and pro-apoptotic effects of DIM.

The IKK/IκB-α/NF-κB pathway is the major molecular mechanism for NF-κB activation. The IKK kinase complex induces phosphorylation of IκB-α at Ser32/Ser36, leading to degradation of IκB-α proteins and resulting in the release of NF-κB; active NF-κB (p65-p50 subunits) is thereby enabled to translocate into the nucleus, where it binds NF-κB-specific DNA binding sites, allowing transcription of downstream survival genes. Our present data show that DIM decreased IκB-α degradation in a dose-dependent manner. Unphosphorylated IκB-α remained bound to the p50-p65 complex, preventing nuclear translocation and activation of NF-κB. We further measured IKKs in the experimental cancer cells. p-IKK-β expression was found to be decreased following DIM treatment, indicating that DIM may inhibit the activation of IKK-β and thus reduce IκB-α phosphorylation and degradation in CNE-2 cells.

In this study, we found that biological effects of DIM occurred at 15–30 *μ*M, but most effects on signaling events required much higher (50 *μ*M) concentrations, suggesting that upregulation of pro-apoptotic molecules and downregulation of anti-apoptotic molecules may partially contribute to DIM-induced apoptosis in cancer cells. Other pathways may also mediate the biological effects of DIM in CNE-2 cells.

In conclusion, DIM may inhibit cell proliferation and induce the apoptosis of CNE-2 cells by regulating multiple molecules in a mitochondria-dependent pathway. Although all of the experiments here were performed in only one cell line and further studies are needed, our results open a new avenue and challenge the current paradigm for the prevention and/or treatment of nasopharyngeal carcinoma. Following up on these points, the effectiveness of DIM in the treatment of NPC is worth further study.
